# A digital microfluidic approach to increasing sample volume and reducing bead numbers in single molecule array assays†

**DOI:** 10.1039/d4lc01002g

**Published:** 2025-02-24

**Authors:** Alinaghi Salari, Jose Gilberto Camacho Valenzuela, Nguyen Le, Joshua Dahmer, Alexandros A. Sklavounos, Cheuk W. Kan, Ryan Manning, David C. Duffy, Nira R. Pollock, Aaron R. Wheeler

**Affiliations:** a Department of Chemistry, University of Toronto 80 St. George Street Toronto ON M5S 3H6 Canada aaron.wheeler@utoronto.ca; b Terrence Donnelly Centre for Cellular & Biomolecular Research, University of Toronto 160 College St. Toronto ON M5S 3E1 Canada; c Institute of Biomedical Engineering 164 College St Toronto ON M5S 3E2 Canada; d Quanterix Corporation 900 Middlesex Turnpike Billerica MA 01821 USA; e Boston Children's Hospital 300 Longwood Ave Boston MA 02115 USA

## Abstract

We report methods that improve the manipulation of magnetic beads using digital microfluidics (DMF) that can enhance the performance of single molecule array (Simoa) digital protein assays in miniaturized analytical systems. Despite significant clinical and biomedical applications for digital protein detection, the development of miniaturized Simoa systems has been limited by the requirements for use of large sample volumes (∼100 μL) and low numbers of beads (∼5000) for high sensitivity tests. To address these challenges, we improved the integration of DMF with Simoa-based assays by developing strategies for loading mixtures of sample and beads into DMF networks using methods relying on either virtual channels or small liquid segments that were applied either in parallel or in a stepwise manner. We have also demonstrated a dedicated densifying electrode technique that captures low numbers of beads within a droplet, allowing high bead retention with minimal residual volumes of liquid. Based on these improvements, we optimized the front-end assay processing of beads using DMF and demonstrated a method to detect tumor necrosis factor α (TNF-α) by Simoa that showed equivalent performance to a microtitre plate assay. The new strategies described here form a step toward integrating DMF and Simoa for a wide range of applications.

## Abstract 2

Digital microfluidics enables processing of large sample volumes (up to 100 μL) with low bead counts (as few as 5000) in single molecule array assays.

## Graphical abstract

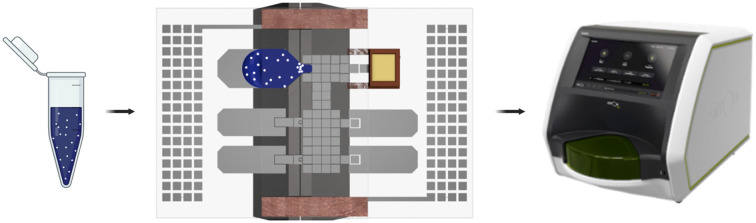

**Tribute to George Whitesides**

A. R. W. fondly remembers George's tenure as Editor-in-Chief of *Lab on a Chip*. Each year at the editorial board meeting, George would pose thought-provoking questions, asking each member for their input. George would then take a strong position on one side of the issue or the other, leading to robust discussions. In subsequent years, he often posed similar questions, and it was not unheard of for him to defend the opposite position the second time around! These conversations were formative for the young journal that owes a tremendous debt to Whitesides' influence.

D. C. D. writes: to me, George M. Whitesides isn't just a world-renowned scientist, he's a scientific ecosystem that has been a primary influence on my career and life. I spent a year as a post-doc in George's lab, and one morning I found a paper left on my desk overnight by him describing an early attempt at PDMS microfluidics. This nudge catalyzed our own efforts in making these devices that helped kickstart a field and took me into miniaturized bioanalytical systems. George's memorably unrestrained criticism of my manuscripts helped my writing immensely: my use of the passive voice was particularly painful to him. Every job I have had since has had some link to the GMW ecosystem. I regularly tell my children they would not exist if it wasn't for Professor Whitesides, he being the reason that I decamped from England to the USA. My abiding memory of George, however, is him and Barbara bopping to “Jump Around” by House of Pain at my wedding; besides being a scientific Leviathan, George is tremendously fun. Thank you, George.

## Introduction

Biomarker detection plays a crucial role across a wide range of research and diagnostic fields, including environmental sciences^1^ and clinical medicine,^2^ and the effectiveness of a given detection system relies heavily on its analytical sensitivity. Among the various detection methods for protein biomarkers, digital immunoassays based on single-molecule arrays (Simoa) have emerged as a highly sensitive approach capable of detecting analytes at ultralow concentrations, reaching sub-femtomolar levels.^3–9^ In Simoa-based digital immunoassays, superparamagnetic microbeads coated with capture antibodies are used to selectively capture protein molecules.^7,10^ Following the incubation of the beads with detection antibodies and an enzyme conjugate, enzyme-labeled sandwich immunocomplexes are formed on the beads. The beads are then isolated in an array of femtoliter-sized microwells containing fluorogenic enzyme substrate, where each bead can be individually sealed into a well and imaged. The digital nature of this method allows for the counting and analysis of signals originating from the presence of individual enzyme-labeled protein molecules. When the number of enzyme-labeled molecules is lower than the number of beads, the molecules distribute according to a Poisson distribution, allowing single molecule quantification.^3,11^

The ultrasensitive nature of Simoa has made it popular for core-lab analysis in hospitals for the detection of low-concentration analytes like tumour necrosis factor alpha (TNF-α), a regulator of inflammatory response and an important biomarker for the pathogenesis of inflammatory diseases.^12,13^ Extension of Simoa to other applications, however, has been constrained by the reliance on large, costly, and complex instrumentation. Microfluidic platforms offer the potential for the development of smaller, simpler, more cost-effective devices for performing Simoa assays, thereby facilitating broader accessibility and utilization of the technology in various diagnostic settings. For example, Lammertyn and coworkers^14–16^ pioneered the use of digital microfluidic (DMF) devices patterned with integrated microwell arrays to implement digital immunoassay measurements on-chip. A fully miniaturized, integrated Simoa platform (relying on DMF or other forms of microfluidics) would likely find many interesting use-cases, for example, in applications outside of the laboratory.

The initial reports^14–16^ of using DMF for Simoa assays represent an important step toward integrated, portable analysis, but several important sample-processing challenges remain to be solved. One key sample-processing challenge is related to bead number. It is known^3,17^ that the sensitivity of Simoa analyses is usually improved by reducing the number of beads. Briefly, on one hand, when bead numbers are reduced, the number of analyte molecules per bead (the relevant parameter for Simoa detection) increases, providing increased sensitivity. On the other hand, capture efficiency decreases when bead numbers are reduced, that works against the improvement in sensitivity. Considering both trends, it has been shown^17^ that 5000–10 000 beads is optimum for high-sensitivity measurements. Unfortunately, the DMF/Simoa applications described previously^14–16^ used large numbers of beads. The use of high bead numbers is typically a requirement for applications in DMF because it helps with bead retention: the magnetic force acting on a pellet of beads scales with the volume of the pellet, and a minimum of 10^5^–10^6^ beads is typically needed to form a pellet that can be retained by magnetic fields for solution exchange.

A second sample-processing challenge that has not been addressed previously is sample volume – for extremely dilute samples, there simply are not enough analyte molecules to detect in small-volume samples.^18^ For example, a 1 μL sample that contains an analyte at attomolar concentration (*i.e.*, 10^−18^ mol L^−1^) contains < 1 molecules. In such cases, a larger sample is needed – *e.g.*, a 100 μL volume of the same sample contains ∼60 molecules of analyte, enough to be detected with ultrasensitive analysis techniques. Unfortunately, DMF is typically not compatible with such large volumes. For example, in a conventional DMF device of the kind used here, the “unit volume” that covers a driving electrode is ∼1 μL, too low for detection of analytes present at trace concentrations.

Here, we introduce solutions to the two challenges outlined above. First, we report a bead-densifying-electrode method that allows for the recovery of small numbers of beads in digital microfluidic devices. Second, we report a new technique to load large sample volumes into digital microfluidic devices. We describe how to combine these methods and to automate them for high-efficiency bead recovery for Simoa analysis and conclude with a demonstration of DMF-Simoa bead processing method for the detection of TNF-α. The results presented here suggest that the integration of DMF and Simoa holds potential to enable miniaturized systems for digital protein detection for a wide range of applications.

## Experimental

### Reagents and materials

The standard dilution buffer used was phosphate-buffered saline (PBS) containing 0.01% (w/v) Tetronic 90R4 surfactant (BASF Corp., Germany), unless otherwise specified. In droplet splitting experiments, deionized (DI) water containing 0.01% or 0.1% (w/v) Tetronic 90R4 was used. In on-chip pelleting experiments, DI water containing 0.5% (w/v) Tetronic 90R4 was used. In all experiments, Simoa superparamagnetic beads (2.7 μm nominal diameter, Quanterix Corporation, USA) were used.

### Device fabrication and operation

Unless otherwise stated, the fabrication, assembly, and operation of the DMF devices followed standard protocols described elsewhere.^19,20^ Briefly, the DMF devices consisted of a glass bottom plate (50.8 mm × 76.2 mm) with a patterned electrode layer made of chromium, and an unpatterned indium tin oxide (ITO)-coated top plate (25 mm × 75 mm). Bottom plates were fabricated using standard photolithography and wet etching at the CRAFT facilities located at the University of Toronto. Two device designs were produced. Design-1 included an array of 57 standard electrodes (∼2.2 mm × ∼2.2 mm), three dispensing electrodes (∼4.3 mm × ∼2.1 mm), one waste collecting electrode (∼4.3 mm × ∼2.1 mm), four reagent reservoir electrodes (∼5.7 mm × ∼14.3 mm), one sample reservoir electrode (∼9.4 mm × ∼16.6 mm), and two right-angled triangular electrodes (with legs of ∼3.4 mm × ∼2.8 mm). Design-2 included an array of 68 standard electrodes (∼2.2 mm × ∼2.2 mm), eight dispensing electrodes (∼4.3 mm × ∼2.1 mm), and eight reservoir electrodes (∼6.7 mm × ∼16.4 mm). In addition, both designs featured bead-densifying electrodes designed for capturing magnetic beads. These electrodes were typically circular (1 mm diameter) and were patterned such that they were positioned between two standard electrodes or occupied a cutout inside one standard electrode. Patterned bottom-plates were then coated with parylene C by chemical vapour deposition (∼6 μm) and FluoroPel PFC 1101V (Cytonix LLC, USA) by spin-coating at 2000 rpm for 30 seconds. Top plates were also coated with FluoroPel by dip-coating, and two layers of double-sided tape (3M Company, USA) were used as spacers to assemble each device, resulting in a separation of ∼190 μm between the plates. The DMF devices were operated using a custom/modified version of the open-source DropBot (Sci-Bots Inc., Canada) actuation system similar to the MR Box v2 reported in Knipes *et al.*^21^ A magnetic lens described previously^21,22^ was positioned beneath the bottom plate and was raised and lowered to densify or release the beads, respectively. In brief, the lens consisted of a neodymium magnet bar flanked by two steel machined arms serving as field directors. A custom stepper motor-controlled Z-stage was employed to activate the lens, raising it nearly to the point of contact with the bottom surface of the DMF device. The movement of the droplets, as well as the activation and deactivation of the magnetic lens, was controlled using MicroDrop software.^23^

### Sample loading strategies

Three strategies were investigated for loading of large-volume liquid samples into DMF. In these experiments, each “sample” was a 100 μL aliquot containing ∼5000 Simoa superparamagnetic beads in PBS supplemented with 0.01% (w/v) Tetronic 90R4.

In the ‘passive’ loading method, the sample was loaded onto the sample reservoir electrode of design-1 devices. By sequentially energizing the electrodes, the sample was drawn into the space between DMF top and bottom plates and moved over the magnetic lens located beneath the densifying electrode. The liquid formed a column of fluid (*i.e.*, virtual channel^19^) stretching across the DMF device, and liquid waste (*i.e.*, sample without beads) was continuously collected into the absorptive media in the waste chamber (see details below). The retained liquid on the densifying electrode and the waste liquid were then imaged using a microscope for bead-capturing analysis. In the ‘parallel’ loading method, the sample was divided into four 25 μL sub-volumes off-chip prior to dispensing into Design-2 reservoir electrodes. These sub-volumes were introduced into the DMF in two subsequent steps, with two sub-volumes loaded simultaneously through two reservoirs. In the ‘stepwise’ sample loading method, the entire sample was dispensed onto the sample reservoir electrode of design-1 devices and pulled toward the waste collection area (as in the ‘passive’ method). But uniquely in this method, just before the advancing front of the virtual channel reached the waste collection area, the volume in the virtual channel (approximately 9 μL) was split from the sample reservoir to form an isolated waste droplet. This waste droplet was then separately collected, while the remainder of the sample was stationary. This process was repeated ∼12 times until the entire sample was loaded.

### Capacitance measurement

The built-in functionality of the DropBot system was employed to measure capacitance of the actuated electrodes, *via* the MicroDrop software,^23^ in the stepwise loading strategy. Briefly, capacitance values were recorded in real-time mode at approximately 40 measurements per second. Initially, when no liquid was present between the top and bottom plates, the system measured a baseline capacitance value that was relatively low due to the air (a medium with low dielectric constant) filling the gap between top and bottom plates. As the liquid entered the gap, the capacitance measured by the instrument increased, reflecting the amount of liquid present on the actuated electrodes. For example, for every ∼9 μL of liquid driven into the gap, the capacitance increased from 100 pF (when the virtual channel was partially created, covering electrodes up to the magnetic lens location) to 250 pF (when ∼9 μL of liquid had passed the magnetic lens location), and then to 320 pF (when the splitting occurred, forming a ∼9 μL droplet). When the liquid was removed from the waste collection area, the capacitance decreased back to its baseline value of 100 pF. To determine the appropriate time for switching to the next step, we introduced a capacitance stability index defined as the rolling standard deviation of the last 15 capacitance measurements, monitored using a custom-written Python script. A low capacitance stability index indicated that the capacitance had stabilized, signifying that the actuated electrode array was fully covered by the liquid. The script defined three sub-steps: (i) load, (ii) split, and (iii) waste removal. The transition between sub-steps was controlled by active feedback – in each sub-step, after an initial increase in capacitance, the next sub-step was triggered when the capacitance stability index fell below a threshold of 2 pF.

### Waste extraction

Whatman 1, 2, 3, 4, and 42 filter papers (Whatman, UK), KimWipes (Kimtech, USA), and the superabsorbent polymer (SAP) sodium polyacrylate (Sigma Aldrich, Canada) were used to extract liquid waste from the DMF device. According to the manufacturer, Whatman 1, 2, 3, 4, and 42 have thicknesses of 180 μm, 190 μm, 390 μm, 210 μm, and 200 μm, and pore sizes of 11 μm, 8 μm, 6 μm, 25 μm, and 2.5 μm, respectively.

A chamber was designed (Fig. S1†) and printed with Clear V3 resin (Formlabs, USA) on a Form 3+ printer (Formlabs, USA) using the stereolithography (SLA) method. To assemble the waste chamber onto the design-1 DMF device, a wick (*i.e.*, a 7 mm × 11 mm section of Whatman 1 or KimWipe) was placed inside the narrow opening at the bottom of the chamber, prior to filling the chamber with 10–40 mg SAP. The chamber filled with SAP was then integrated into the DMF device such that the wick was positioned on the waste collecting electrode, penetrating the space between the top and bottom plates (Fig. S2a†).

In experiments using only filter paper (without SAP), the waste chamber was not employed (on design-1 devices). Instead, either a single layer of paper that served as the absorbent and the wick (a quadrant with a radius of 60 mm) or a stack of 15 layers of paper [7 mm × 11 mm each, except for the bottom one (the wick) which was 7 × 16 mm] was used. In experiments with a single layer, approximately 5 mm of the paper tip was positioned on the waste collecting electrode penetrating the space between the top and bottom plates, while the remaining of the paper extended outside (Fig. S2b†). Note that the nominal thicknesses of some of the filter papers used were greater than the inter-plate spacing. Thus, the tips of those papers were excised from the lateral side using a blade to achieve the desired reduction in thickness, allowing them to fit within the pre-defined gap. Similarly, in experiments with a stack of multiple layers, the wick was positioned such that it penetrated the space between the top and bottom plates, while the remaining of this paper extended outside. The other 14 layers of papers were then stacked on top of the wick (Fig. S2c†).

### Bead extraction and counting in small-volume samples

In experiments characterizing bead retention in small sample volumes, 4 μL aliquots of suspensions of ∼100–5000 Simoa superparamagnetic beads were mixed with 0.01% (w/v) Tetronic 90R4 in PBS. To estimate the expected number of beads, stock suspensions with various bead counts were first prepared, ensuring that each 1 μL of the stock contained the specified number of beads. The number of beads in a 1 μL droplet from each stock suspension was counted and then diluted to 4 μL before being loaded onto a design-1 device. In experiments evaluating bead retention on densifying electrodes, the 4 μL droplet containing the beads was moved across the magnetic lens while the densifying electrode was activated. To find the number of beads in the waste droplet, the waste liquid segment was inspected for the presence of beads, using microscopy. Bead retention percentage was then calculated as [1 − (bead count in the waste droplet/expected number of beads)] × 100.

### Bead extraction and counting in large-volume samples

In experiments evaluating the number of beads collected on the densifying electrode from large sample volumes, 100 μL aliquots containing ∼5000 Simoa superparamagnetic beads in PBS supplemented with 0.01% (w/v) Tetronic 90R4 were dispensed onto the sample reservoir in either design-1 or design-2 device and then loaded using one of the three methods described above. Once bead densification was completed, the droplet containing the densified beads—either as-is or after being merged with a 1–2 μL droplet of PBS (supplemented with 0.01% 90R4)—was mixed on-chip to disperse the beads prior to imaging and counting.

To estimate the expected number of beads in a 100 μL sample (at a bead density of 50 beads per μL), either 2 μL aliquots of the corresponding bead suspension or 1 μL of the stock suspension (at a bead density of 5000 beads per μL) were loaded onto the DMF device (without densification) and counted immediately. The expected number of beads was extrapolated from the average count in the first case and calculated as the average of the beads counted in the second case, respectively. Bead retention percentage was then calculated as [bead count in the densified droplet/expected number of beads] × 100.

### Bead imaging and analysis

In all experiments, devices were imaged using a Nikon upright Eclipse (Ni-E) microscope, equipped with an SCMOS camera. Brightfield images were acquired at 20× magnification with an exposure time of 200 μs and then analyzed to determine the bead counts using either a custom-written MATLAB script (MathWorks, USA) or by manual inspection.

### Liquid retention

In experiments aimed at evaluating the reliability of liquid retention on densifying electrodes, 4 μL or 4.5 μL droplets were loaded onto design-1 or design-2 DMF devices. In each experiment, the magnetic lens was engaged, and the droplet was translated across it for two types of experiments (‘pelleting’ and ‘densifying electrode’). In ‘pelleting’ experiments, the densifying electrode was not activated during this process. A pelleting event was determined to be successful when an aggregate of beads remained behind as the droplet moved away from the magnet. Conversely, if the beads moved along with the liquid during the droplet's passage, it was categorized as unsuccessful. In densifying electrode experiments, the densifying electrode was activated during droplet transport. The volumes that were retained (for those that were successful) were estimated by extrapolating from the droplet area, given the known inter-plate gap.

### Characterizations of the bead-densifying electrodes

In experiments evaluating the shape and size of the densifying electrodes, round and elliptical electrodes were formed in design-2 devices. Round electrodes had diameters of 0.6, 0.8, 1.0, or 1.2 mm. Elliptical electrodes had a major axis of 2.4 mm, with minor axes of 0.6, 0.8, 1.0, or 1.2 mm. In the designs, the round or elliptical bead densifying electrodes were positioned either between two standard electrodes or within one standard electrode. A mathematical model (Note S1 in the ESI†) was used to further characterize the effect of electrode size and shape on the bead densification process. The derived equations were solved and plotted using MATLAB (MathWorks, USA).

### Simoa bead processing

Simoa superparamagnetic beads coated with anti-TNF-α capture antibodies, TNF-α antigen, biotinylated anti-TNF-α detection antibody, streptavidin-β-galactosidase (SβG), resorufin-β-d-galactopyranoside (RGP), wash buffers, and diluent buffers, were obtained from Quanterix Corporation, USA. Each reagent provided as a solution was supplemented with 0.1% (w/v) Tetronic 90R4. The equipment used for running the assay in a microtiter plate and imaging the beads included a plate shaker, plate washer, and SR-X imager system (Quanterix Corporation, USA). In DMF processing, a 100 μL sample (solution of TNF-α), a 25 μL suspension of 15 000 functionalized beads, and a 20 μL aliquot of detection antibody were initially mixed and incubated for 20 min off-chip on a shaker at 800 rpm. Following sample incubation, the remaining steps, including bead densifying, detection antibody and SβG incubations, and washing steps, were conducted on a design-1 DMF device. Upon completion of the DMF process, the processed bead suspension was collected by pipette and transferred to a 96-well microtiter plate for analysis by the SR-X, where it was analyzed according to manufacturer's instructions. Briefly, the processed bead suspension was loaded into microwells, sealed with oil, and subjected to imaging and analysis to determine the average number of enzyme labels per bead.^11^ In control experiments, all incubation and mixing steps were performed in a 96-well microtiter plate as described elsewhere.^3^

## Results and discussion

**Fig. 1:**
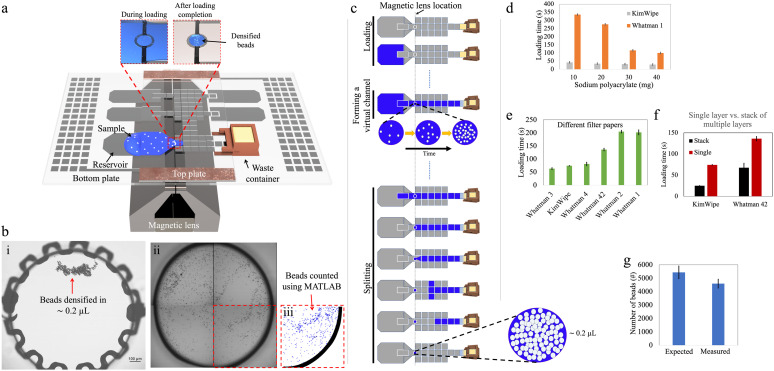
Concept of passive sample loading and densifying of Simoa beads on DMF. (a) Schematic representation of an assembled DMF device (‘design-1’) with a magnetic lens. A large ‘sample reservoir’ accommodates the entire 100 μL sample volume for bead densifying. A circular densifying electrode is positioned in the path between the sample reservoir and a waste chamber. The two insets show bead densification during (left) and after (right) the completion of loading. (b) Beads are counted in three steps, illustrated by (i) an image of beads after densifying on the device, (ii) an image of beads collected from a device and imaged by brightfield microscopy, and (iii) the output of a custom MATLAB script quantifying the number of beads observed (outlined in blue). (c) Schematic illustrating the workflow of sample loading (top), formation of a virtual channel (middle) and bead recovery on the densifying electrode (bottom). (d–f) Plots of loading time (defined as the duration required to load 100 μL sample into the DMF device) as a function of the material in the waste chamber, including (d) superabsorbent polymer (sodium polyacrylate) with wicks formed from KimWipe (gray) or Whatman 1 (orange), (e) single-layer paper (green), and (f) stacks of 1 (red) or 15 layers of paper (black). (g) Plot of expected (left) and measured (right) numbers of beads retained when 25 mg of sodium polyacrylate with a KimWipe wick was used for waste extraction. Error bars represent mean ± standard error (in d, *n* = 3; in e and f, *n* ≥ 3; in g, *n* = 5).

**Fig. 2:**
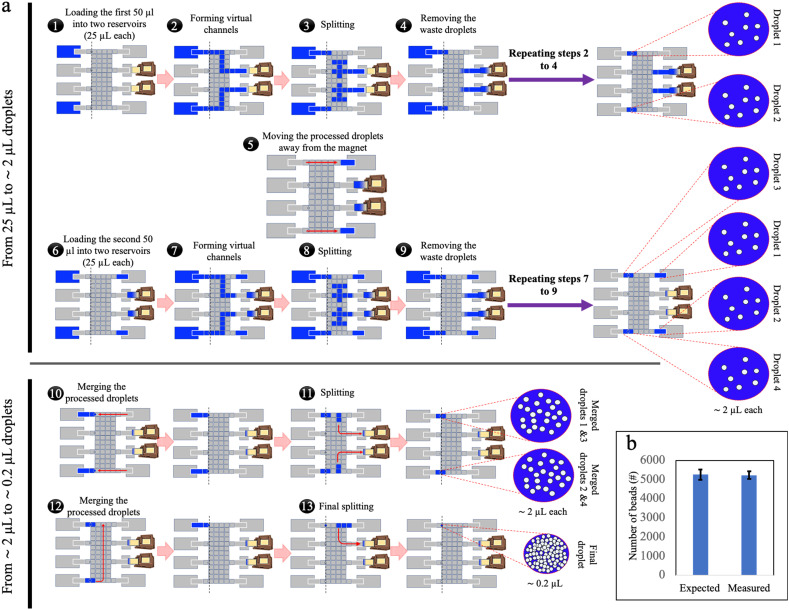
Parallel loading of sub-volumes of sample-bead mixtures onto DMF. (a) Schematic representation of parallel loading of sample sub-volumes onto two (out of the four available) processing lines of electrode arrays on a design-2 DMF device. Each processing line handled 25 μL of sample, resulting in two ∼2 μL droplets (steps 1–9). Once four processed droplets were formed, they were merged, and the bead-densifying process was repeated to obtain a final droplet of ∼0.2 μL containing all the beads (steps 10–13). The dashed lines indicate the location of the magnetic lens when the magnet was engaged. (b) Plot of expected (left) and measured (right) numbers of beads retained using the parallel loading strategy. Error bars represent mean ± standard error (*n* ≥ 4).

Our goal for this work was to explore the use of DMF for paramagnetic bead processing upstream of analysis by Simoa to enable the use of large sample volumes (∼100 μL) that contained low bead numbers (∼5000 beads). Fig. 1 and 2 show two DMF devices, ‘design-1’ and ‘design-2’, respectively, that were developed for these studies. In many of these experiments, the “samples” were simply buffers that were manually mixed with beads (off-chip) to attain the desired density. In future work, new methods might be developed to automate the process of bead dispersion in samples to allow for hands-free operation.

### Manipulation of small bead numbers using DMF

We first explored the ability for DMF to fluidically manipulate small numbers of magnetic beads that result in the most sensitive Simoa assays.^3^ The first step after the sample-bead mixture enters a DMF device is to separate the beads from the liquid. The conventional approach in a DMF device is a ‘pelleting’ technique, in which a magnetic field is engaged to pull the beads into an agglomerated pellet on the surface of the device, while the fluid is pulled away.^20^ The advantage of the pelleting technique is that the beads are recovered in a very small volume; the disadvantage of the pelleting technique is that the magnetic force must be strong enough to keep the pellet stationary, resisting the interfacial force acting on the pellet by the departing droplet. In practice, the large interfacial forces involved means that large numbers of beads must be used, to increase the size and magnetic susceptibility of the pellet. In initial experiments using the devices described here (Table S1†), we found that ∼0.5–1.0 × 10^6^ beads could be pelleted, whereas smaller quantities could not.

To overcome the limits of the pelleting technique, we developed a method using a ‘densifying electrode’, to hold the beads temporally in a relatively small droplet. The densifying electrode was a designated electrode in the device that was designed to remain ‘on’ during loading of the sample-bead mixture, such that when the magnet was engaged, the magnetic force held the beads on the densifying electrode while the attractive force generated by the actuated electrode resisted the interfacial force applied by the departing droplet. Note that this approach is conceptually similar to the “one-to-three” technique described by Jin *et al.*^24^

Fig. 1a illustrates an assembled design-1 device bearing such an electrode, and Fig. 1b shows how beads can be recovered by this strategy. Initial experiments (Fig. S3†) demonstrated that it was possible to reliably retain and recover as few as 100 beads on the densifying electrode with high efficiency (∼98%), representing orders of magnitude fewer beads relative to the smallest number of beads that could be captured by the pelleting technique. On the other hand, this approach sacrifices the advantage of tiny recovery volumes, as fluid is retained on the electrode with the densified beads. By making the densifying electrode small, this volume can be minimized, as described in detail below.

### Manipulation of large sample volumes loaded into DMF devices

We then investigated several methods to load large sample volumes onto the device for bead extraction. A standard sample volume of 100 μL containing 5000 beads (as a proxy for what is needed in a high-sensitivity Simoa experiment) was used to evaluate three different loading techniques: ‘passive’, ‘parallel’, and ‘stepwise’ loading. For the current application, the ‘stepwise’ method was ultimately selected; however, we speculate that the ‘passive’ and ‘parallel’ methods may find use-cases in other applications that have different requirements.

### Passive loading of high-volume samples

In initial work, inspired by the previously introduced P-CLIP technique,^19^ a ‘passive loading’ method was developed in which a virtual channel was formed to connect the ‘source’ (the sample containing suspended beads) with a ‘sink’ (an absorbent solid in the waste chamber). As the fluid was absorbed into the solid, the fluid was passively pulled across the device so that the beads could be collected at the densifying electrode (Fig. 1c).

In the passive loading technique, the rate at which liquid enters the DMF device and forms a virtual channel is mainly governed by the applied DMF forces. Once the liquid reaches the absorbent material in the waste collection area and throughout the remainder of the loading process, the flow rate is determined by the liquid-absorption process. Faster extraction of waste leads to shorter sample loading times, thereby reducing assay times. Uncontrollably fast loading can, however, lead to poor bead retention as described below. To evaluate and optimize this process, we tested different materials and geometries.

We first tested the use of sodium polyacrylate—a superabsorbent polymer (SAP)—as the absorbent material in the waste collection area to drive the passive loading process. We designed a 3D-printed waste collection chamber (Fig. S1†) to accommodate different amounts of SAP. A paper wick positioned at the bottom of the chamber connected the SAP with the waste liquid in the DMF to allow for efficient fluid transport (Fig. S2a†). Once assembled, approximately 5 mm of the paper wick penetrated the inter-plate space in the DMF device, and the bottom of the chamber was sealed to prevent SAP expansion against the bottom plate. This design facilitated off-chip refilling of the chamber and easy integration with the DMF device, reducing friction between the chamber and the electrode surface and potentially enabling multiple uses of the container. In this setup, wicks formed from KimWipe and Whatman filter papers (which are commonly used in paper-based microfluidics^25,26^) were used, because of their availability in different thicknesses and pore sizes. As shown in Fig. 1d, increasing the mass of SAP in the chamber from 10 to 40 mg shortened the loading time. At the extreme, when using a KimWipe wick with 40 mg of SAP, the loading time for a 100 μL sample was ∼30 s.

As an alternative to SAP, we also explored the use of single layers of paper that served both as ‘wick’ and the absorber (Fig. S2b†). Fig. 1e shows that the loading time for this type of system decreased with thicker papers. For example, the loading time can be as short as ∼1 min for Whatman 3 paper (nominally 390 μm thick) and more than ∼3 min for Whatman 1 paper (nominally 180 μm thick). We also explored using stacks of 15 layers of paper (Fig. S2c†). As shown in Fig. 1f, the loading time was roughly halved for a 15 ply stack of Whatman 42 papers and reduced to one-third for a 15 ply stack of KimWipes, compared to their corresponding single-ply absorbers. This behavior can be explained by Darcy's law that describes the flow rate of a fluid through a porous medium; a similar trend was reported previously for P-CLIP.^19^

An important drawback that was observed for the ‘passive loading’ technique was the variation of flow rate within the virtual channel during waste extraction. This variation in flow rate resulted in different levels of drag force experienced by beads suspended in the virtual channel over the magnetic lens, and, in some cases, the drag force exceeded the opposing component of the magnetic force, leading to undesirable bead loss. For instance, when using the waste chamber filled with 25 mg of SAP, bead loss of ∼15% was observed upon loading (Fig. 1g). The observed bead loss highlighted the importance of carefully considering the flow dynamics and drag forces within the virtual channel when designing the waste extraction method in DMF systems. We therefore sought to develop a method with greater control over the flow of sample-bead mixtures in these devices.

### Parallel loading of high-volume samples

To address the challenge of varying flow rates (and their effect on bead retention) within the virtual channel in passive loading, a ‘parallel loading’ strategy was developed using a larger DMF network (design-2), where sub-volumes of sample were loaded in parallel (Fig. 2a and Video S1†). Specifically, in this method, prior to dispensing, the initial 100 μL sample was split off-chip into four 25 μL sub-volumes that were small enough to ensure complete loading into the DMF network without the need for simultaneous waste extraction. In the parallel-loading strategy, each sub-volume can be processed separately, and the isolated waste droplets can be collected by an absorber independently during loading or as a subsequent step. By breaking the virtual channel before it reaches the waste collection area, the flow rate during bead retention was primarily governed by the DMF forces that can be kept manageably low. The method demonstrated excellent bead retention: when samples containing 5272 ± 269 beads (ave. ± std. error) were loaded, an average of 5240 ± 191 beads (ave. ± std. error) were recovered, a retention rate of more than 99% (Fig. 2b), compared to the ∼85% bead loss in passive loading. This strategy, however, required dedicating a large area of the chip solely to sample loading, increasing the device size and complexity, and fabrication costs. In addition, the requirement that the sample be divided into four sub-samples prior to loading into the device is not optimal from a user-perspective.

### Stepwise loading of high-volume samples

**Fig. 3:**
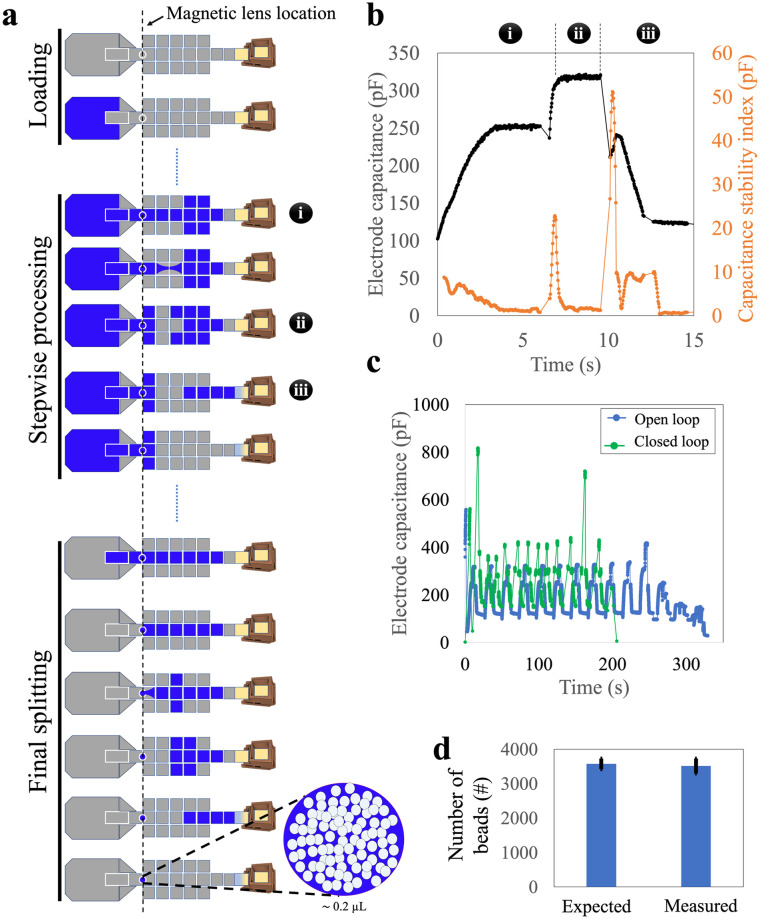
Stepwise loading of the sample onto DMF. (a) Schematic representation of the workflow, comprising dispensing of the sample into the reservoir (top), iterative, stepwise, handling of ∼9 μL segments of sample [middle, with repeating cycles of sub-steps (i) loading, (ii) splitting, and (iii) delivery to waste], and handling of the final segment of sample (bottom). (b) Representative plots of real-time capacitance (black, left axis) and capacitance stability index (orange, right axis) as a function of time for one cycle of sub-steps (i)–(iii). (c) Representative plots of capacitance readings as a function of time for the loading of a 100 μL sample (*i.e.*, approximately 12 cycles) for open-loop (blue) and closed-loop (green) loading processes. (d) Plot of expected (left) and measured (right) numbers of beads retained using the automated stepwise loading strategy. Error bars represent mean ± standard error (*n* = 5).

To address the large device sizes and sub-optimal user experience associated with the parallel loading technique, we developed a third ‘stepwise loading’ method that incorporated the most useful features from the passive and parallel approaches. We used the smaller design-1 devices to test this stepwise method. In this approach, the entire sample was dispensed at once but was processed sequentially (Fig. 3a and Video S2†). Approximately 12 cycles—each comprising three sub-steps, (i) the loading of a ∼9 μL segment of sample, (ii) splitting of that segment (and collecting the beads), and then (iii) waste removal—were needed to load a 100 μL sample into the device.

We faced two key challenges in developing the stepwise loading method. The first challenge was related to the reliability of the splitting sub-step (ii) (splitting) that was observed to change as the cycles were repeated. We attribute this observation to the fact that this sub-step depends on both the DMF force (which does not change from cycle to cycle), as well as hydrostatic and Laplace pressures (which change from cycle to cycle as the sample is used up). In particular, as illustrated in Fig. S4,† the Laplace pressure resisting sub-step (ii) increased as the volume in the sample reservoir decreased, making the second-to-final and final cycles particularly challenging to perform. We addressed this challenge by programming sub-step (ii) such that both ends of the liquid segment were stretched apart, perpendicular to the direction of the necking region, and (in some cases) allowing the final one or two segments to be larger than 9 μL. This process increased the distance between the two segments of the splitting liquid, resulting in a narrower necking region, facilitating reliable splitting from cycle-to-cycle.

The second challenge related to the duration of each sub-step and cycle in the segment-loading process. These durations were found to be inconsistent, primarily due to changes in the volume of the remaining liquid on the reservoir electrode during loading. As a result, a pre-programmed method (with specified durations) often failed, leading us to carry out ‘open loop’ sample loading, where the operator chose in real time when to move to the next sub-step. This open-loop process was slow and not suitable for automated operation. We addressed these issues by sensing the progress of droplet movement on DMF using capacitance measured at each sub-step. Fig. 3b illustrates this approach: each sub-step had a characteristic pattern of capacitance and capacitance stability index. Thus, building on previous reports of capacitance-based ‘closed loop’ automated sample handling,^27^ we developed a closed-loop/automated protocol for this process. Representative stepwise loading (requiring approximately 12 cycles) using the user-controlled, open-loop and the automated closed-loop methods are shown in Fig. 3c. The closed loop process was faster (∼4 *vs.* ∼6 min), and most importantly was automated, allowing for hands-free use.

With closed-loop capacitance control, the stepwise loading method was observed to be robust and reliable, achieving a bead retention of more than 98% (Fig. 3d). This method was fully automated, could be completed within four minutes, and required a smaller and simpler device than the parallel method. We, therefore, used the stepwise method for the remainder of the work described here.

### Recovery droplet volume

As a final optimization step, we explored the process of droplet recovery on the bead densifying electrode. This process can be considered similar to passive dispensing,^28^ in which a large droplet on a DMF device is passed over a small hydrophilic spot, often used for adherent cell culture applications.^29^ This ‘recovery’ volume is important in Simoa assays as it represents the residual volume of a sample or assay reagent during each densifying-liquid removal step. Minimization of this residual volume is important in Simoa to reduce assay backgrounds, and as described previously,^8^ these residual volumes must typically be kept to <1 μL for good assay performance.

**Fig. 4:**
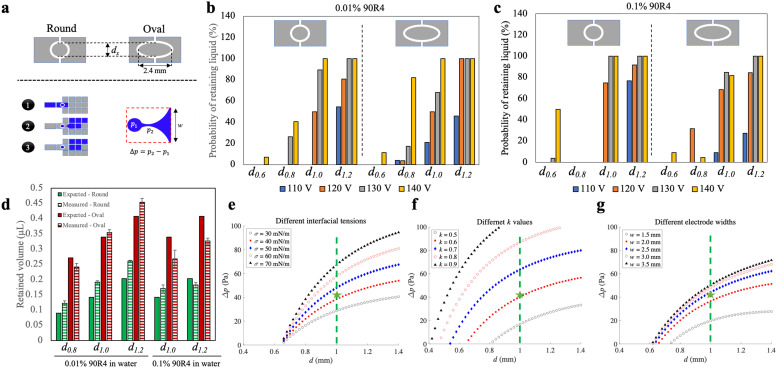
Characterization of droplet retention on the densifying electrode. (a) Schematic representations of (top) two different shapes (round *vs.* oval) of the densifying electrode with diameter *d*_*x*_, (bottom-left) the three-step actuation scheme used in these experiments for a primary destination electrode with width *w*, and (bottom-right) the pressure imbalance Δ*p* for the retained droplet (*p*_1_) relative to the necking region (*p*_2_) connecting the retained and moving droplet. (b and c) Plots showing the probability of successfully generating retained liquid from an initial ∼4.5 μL droplet (for at least 20 replicate measurements in each condition) for different shapes (round-left *vs.* oval-right), sizes of the densifying electrodes (left-to-right: *d*_*x*_ = *d*_0.6_, *d*_0.8_, *d*_1.0_, and *d*_1.2_, where sub-scripts are diameters in mm), and applied voltages (110 V_RMS_-blue, 120 V_RMS_-orange, 130 V_RMS_-gray, and 140 V_RMS_-yellow), for (b) 0.01% 90R4 in water and (c) 0.1% 90R4 in water. (d) Plot of expected (solid, extrapolated from electrode area) and measured (hatched) volumes of retained droplets on round (green) and oval (red) densifying electrodes for 0.01% (left) and 0.1% (right) 90R4 in water. The error bars represent mean ± standard error (*n* = 3). (e–g) Plots of results of the mathematical model predicting Δ*p* as a function of the diameter of round densifying electrode for (e) liquid interfacial tensions *σ* (30-open black circles, 40-filled red squares, 50-filled blue diamonds, 60-open red circles, 70-filled black triangles mN m^−1^), (f) *k* values (defined in Note S1 in the ESI,† 0.5-open black circles, 0.6-filled red squares, 0.7-filled blue diamonds, 0.8-open red circles, 0.9-filled black triangles), and (g) destination electrode widths *w* (1.5-open black circles, 2.0-filled red squares, 2.5-filled blue diamonds, 3.0-open red circles, 3.5-filled black triangles mm). Green dashed lines and green stars in (e–g) indicate the circular *d*_1.0_ electrode used in most experiments, and values assumed to be close to experimental conditions [*σ* = 42 mN m^−1^ in (e), *k* = 0.6 in (f), and *w* = 2.2 mm in (g)], respectively.

We evaluated the probability of droplet retention (and the volume of retained droplets) on densifying electrodes with round and elliptical shapes, and of varying sizes (Fig. 4a-top). An illustration of the process, along with a model of pressure imbalance Δ*p* experienced during the retention process, are shown in Fig. 4a-bottom. Different DMF driving potentials were also tested, and because Δ*p* is impacted by surface tension, two different fluids were evaluated – aqueous solutions of 0.01% and 0.1% of the surfactant 90R4. The results, shown in Fig. 4b and c, indicate that elliptical electrodes had a higher probability of liquid retention on average, but they also resulted in a higher retained volume (Fig. 4d). Likewise, round electrodes with diameters equal to the minor axis of the corresponding elliptical electrodes exhibited a lower retained volume. In general, we found that the probability of liquid retention increased with larger densifying electrode surface area and higher actuation potentials, both of which led to increased retained liquid. This corresponds to an estimated range of ∼15–45 μN in DMF driving force (assuming the validity of Young–Lippmann equation^30–32^ with a parylene layer thickness of 6 μm and a dielectric constant of 3.1) in our experiments. Within this range, the probability of liquid retention increases from 0 to 100% as the DMF force rises from ∼15 to ∼45 μN.

Round densifying electrodes with a diameter of 1 mm or greater at high driving potentials were found to have reliable liquid retention with a small retained volume for both high and low surface tension solutions, so this condition was used in the remainder of the experiments described here. These conditions allow the generation of droplets with a volume of ∼0.2 μL with high reproducibility (Fig. S5†). This volume is similar to what can be achieved through passive dispensing on a hydrophilic spot^28,33^ of the same size as a densifying electrode, but with the added advantage of being dynamic (*i.e.*, the bead densifying electrode can be turned off and on as needed). The retained volume with the densifying electrode is larger than the volume formed by pelleting (Fig. S5†) but still allows for substantial reduction in residual volume. For example, for a 100 μL sample (as described above), beads are recovered in a volume that is reduced to 0.2% of the original volume on the densifying electrode.

We developed a mathematical model (Note S1†) to explore the relationship between the effect of electrode dimensions (driving electrode width *w* and densifying electrode diameter *d*), liquid interfacial tension (*σ*), and contact angle difference [*k*, see eqn (S11) in Note S1†] on the pressure imbalance Δ*p* expected for a droplet being dispensed. In this model, a larger Δ*p* suggests a greater probability of droplet recovery on a densifying electrode. Fig. 4e–g summarizes the effects expected for different parameter values on Δ*p*. The results demonstrated that higher interfacial tensions (corresponding to lower concentrations of surfactant, Fig. 4e) and higher values of *k* (associated with higher applied voltages and lower surfactant concentrations, Fig. 4f) led to higher Δ*p* for any given densifying electrode diameter. However, the increase in Δ*p* was not significant for larger sizes of the electrode array beyond 2.5 mm (Fig. 4g). Among the parameters investigated, our results indicate that a 1 mm round densifying electrode (shown by green dashed lines in Fig. 4e–g) generates a relatively high Δ*p*, corresponding to a reliable droplet breakup. More generally, this model can provide useful insight for the development of future devices and systems relying on densifying electrodes.

### Exploration of low bead-number, large-sample-volume DMF processing for Simoa detection

Previous reports^14–16^ that integrated microwells with DMF for Simoa applications used around 110 000–300 000 beads in droplets with volumes of 1.1–2.5 μL. As state-of-the-art Simoa assays^3^ require the use of significantly fewer beads (∼5000) and greater sample volumes (∼100 μL), the improved methods for performing assay steps on magnetic beads using DMF described here represent substantial advances for the eventual goal of miniaturizing Simoa.

**Fig. 5:**
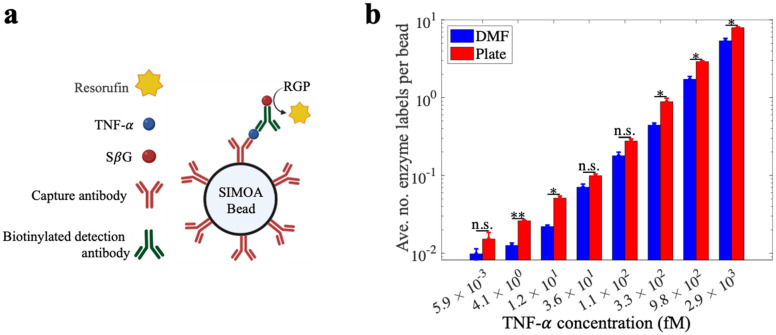
Demonstration of Simoa bead processing scheme using the DMF methods developed in this work. (a) Schematic representation of the assay components for capturing TNF-α (blue) *via* immunocomplexes formed from capture antibody (red), biotinylated secondary antibody (green), and streptavidin-β-galactosidase (SβG, red circles) that catalyzes the formation of resorufin (yellow) from resorufin β-d-galactopyranoside (RGP). The assay processes were performed on DMF and in a well-plate platform, followed by transferring the beads to the SR-X imager for detection. (b) Bar plot of average number of enzyme labels (SβG) per bead as a function of TNF-α concentration for samples processed using DMF (blue) and well-plate (red) methods. Error bars represent mean ± standard error (*n* ≥ 2). Asterisks denote significance for un-paired *t*-test comparisons between DMF and well-plate data: *p < 0.05, **p < 0.01, “n.s.” no significance.

Because of the importance of TNF-α as a biomarker for inflammatory diseases^12,13^ we chose it as a model analyte to evaluate the DMF-Simoa bead processing techniques introduced here (Fig. 5a). Samples were tested using reagents for a Simoa TNF-α assay, where the assay steps were performed using either a microtiter well plate^3^ or using the step-wise loading strategy (Fig. 3) with 1 mm dia. round densifying electrodes, followed by imaging of labeled beads using a modified SR-X imager.^3^ As shown in Fig. 5b, the assay calibration curves obtained by the DMF process and conventional (manual) processes were comparable. That is – the trends of signal as a function of analyte concentration were similar, but there were small differences observed for some of the average number of enzyme labels per bead values. These data demonstrate the viability of using the new DMF-based method to realize a fully automated bead immunoassay process that is amenable to miniaturization.

Finally, we acknowledge that this is a proof-of-concept study, and that future steps will be needed for full integration of DMF with Simoa. For example, as noted above, beads and samples are combined and mixed manually, off-chip. And after processing on-chip, samples were collected manually and then loaded into an SR-X imager for loading and sealing in an array of microwells for analysis. We can envision future versions of this system in which each of these steps is automated on a single platform, perhaps with an on-chip microwell array as has been demonstrated previously.^14–16^

## Data availability

The raw data associated with this manuscript is available from the corresponding author upon request.

## Conclusion

In this work, we have addressed two key challenges associated with the miniaturization of Simoa assays on DMF devices: bead loss at very low bead numbers and the need to load high volumes of liquid samples on DMF platforms, both of which are needed for the highest sensitivity digital assays. By developing and optimizing new strategies for sample loading and a new densifying electrode strategy, a scheme was demonstrated that minimizes bead loss while maintaining the capacity to work with large-volume samples. We developed a mathematical model that supported the design of the densifying electrode that can be applied to other bead-based DMF applications. Finally, the automated microfluidic method had comparable performance to the standard assay processing method in which samples were processed manually by pipettes in a microtiter well plate. We propose that the combination of DMF and Simoa has the potential to become a powerful new technique to automate assay workflows and enhance the sensitivity of biomarker detection.

## Author contributions

A. S. and J. G. C. V. contributed equally to the study. J. G. C. V. and A. A. S. developed the concepts of the loading strategies. A. S. and J. G. C. V. designed, fabricated, and assembled the DMF devices, and also wrote the original draft. A. S., J. G. C. V., N. L. and J. D. conducted and analyzed the DMF experiments. A. S. developed the concept of densifying electrode and carried out the mathematical model. J. D. and J. G. C. V. developed the capacitance-based automated program. C. W. K. and R. M. designed, conducted, and analyzed the TNF-α assay. D. C. D., N. R. P., and A. R. W. defined the overall goals and approach of the work, secured funding for the study, and provided supervision. A. S. and A. R. W. wrote the manuscript, and all authors contributed to the discussion of the results and the conceptualization of the study.

## Conflicts of interest

C. W. K., R. M., and D. C. D. are employees of one of the funders of the project.

## Supplementary Material

LC-025-D4LC01002G-s001

LC-025-D4LC01002G-s002

LC-025-D4LC01002G-s003
