# Supplementary material for: A digital microfluidic approach to increasing sample volume and reducing bead numbers in single molecule array assays
Source: Lab Chip. 2025 Feb 24;25(7):1669–80. doi: 10.1039/d4lc01002g (PMC11849296; doi:10.1039/d4lc01002g)
Supplement: LC-025-D4LC01002G-s001 [file LC-025-D4LC01002G-s001.pdf]

Supplementary Information (SI) for Lab on a Chip.
This journal is © The Royal Society of Chemistry 2025

Supplementary Information for **A Digital Microfluidic Approach to Increasing Sample Volume and Reducing Bead Numbers in Single Molecule Array Assays**

Alinaghi Salari,† Jose Gilberto Camacho Valenzuela,† Nguyen Le, Joshua Dahmer, Alexandros A. Sklavounos, Cheuk W. Kan, Ryan Manning, David C. Duffy, Nira R. Pollock, and Aaron R. Wheeler

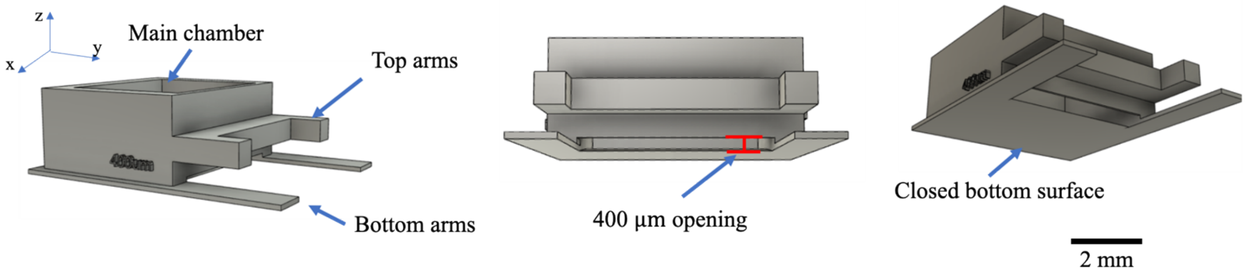

Figure S1. Schematic views from different angles of the 3D-printed chamber used to hold the superabsorbent polymer (SAP) in waste extraction experiments. It includes a main chamber (approximately 7 mm x 6 mm x 4 mm) interfaced with "top arms" (1 mm x 3 mm x 1 mm) designed to extend over the top of the top plate of a DMF device and "bottom arms" (1.5 mm x 5 mm x 0.2 mm) designed to penetrate the gap between the top and bottom plates of a DMF device. A paper wick (not shown) is initially inserted as the bottom layer of the chamber through the 400 µm opening (illustrated in red in the schematic in the middle) before filling the chamber with SAP. The orthogonal vectors indicating the x, y, and z directions refer to the view on the left.

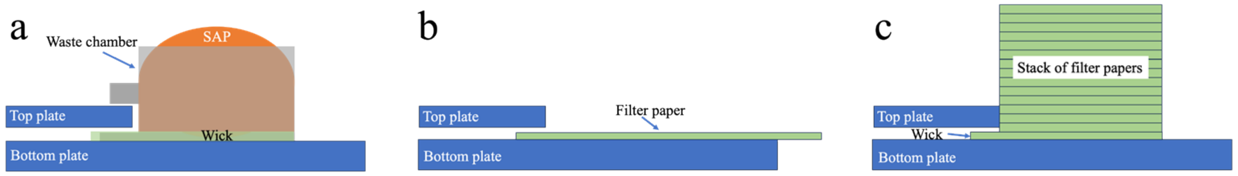

Figure S2. Side-view schematics (not to scale) of the three waste collection methods used in this study. (a) A waste chamber (gray) filled with SAP (orange) and coupled with a paper wick (green). (b) A single layer of paper that serves as the wick and the absorbent. (c) A stack of filter papers. The bottom layer forms the wick.

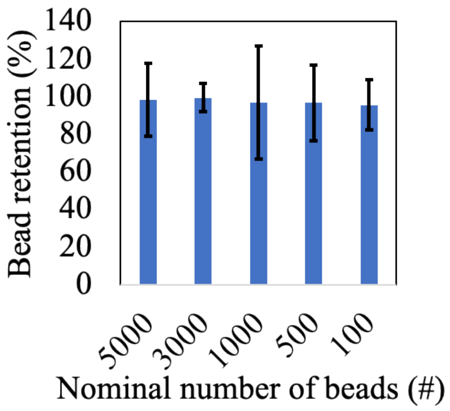

Figure S3. Plot of densifying electrode-driven bead retention for various bead counts in Design-1 devices with 1-mm diam. circular densifying electrodes. Error bars represent mean ± standard error (n = 3). In these experiments, 130 V_{RMS} was used to move 4 µL bead suspension droplets in 0.01% 90R4 in PBS.

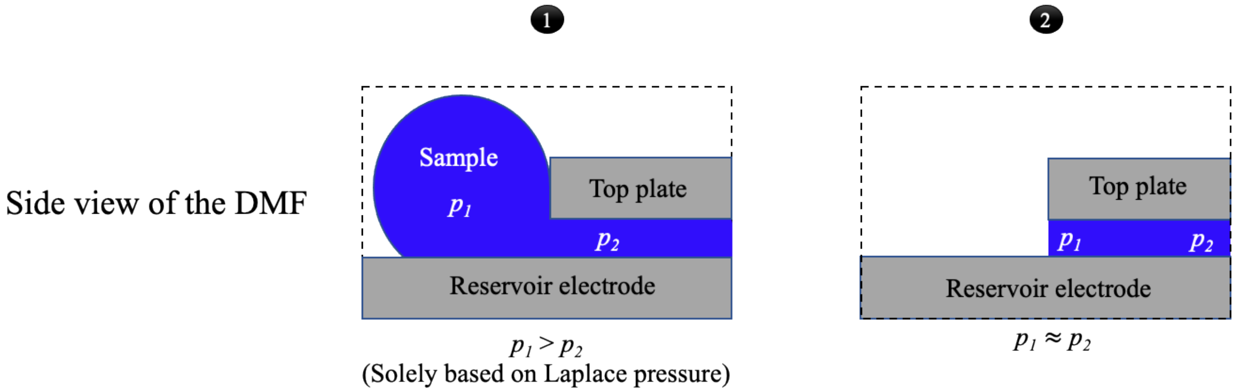

Figure S4. Side-view schematics of the sample reservoir at the DMF top plate edge, illustrating the critical step of liquid entry into the DMF gap. Time points 1 and 2 represent the moments just before and after the liquid enters the device, respectively. At time point 1, the Laplace pressure undergoes a significant increase, causing the liquid entry rate to exceed the waste extraction rate. This can result in the liquid spilling over the electrodes adjacent to those dedicated to the virtual channel. To address this effect, activation of additional electrodes (those not initially used for the virtual channel) is required at this step to maintain control over the liquid flow.

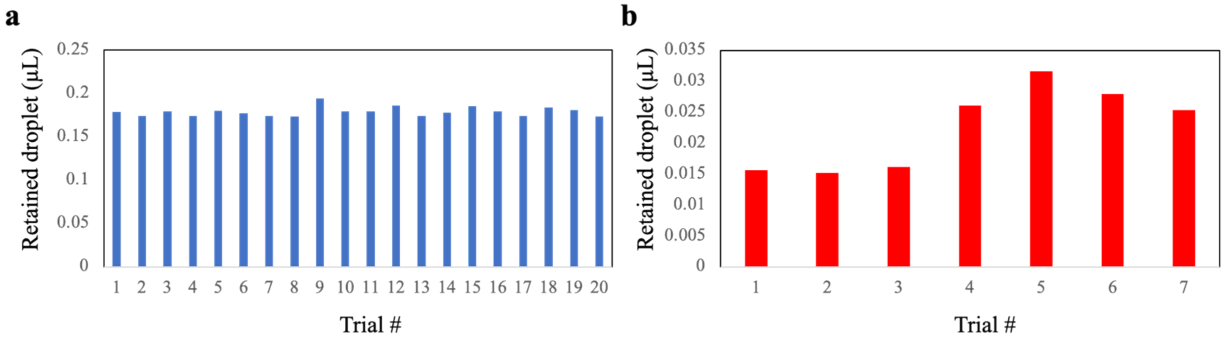

Figure S5. Plot of volume of retained liquid for repeated bead retention experiments for (a) densifying electrode and (b) pelleting experiments. In densifying electrode experiments (blue bars), a droplet containing a low number of beads (fewer than 5,000 beads) was moved over the 1-mm round densifying electrode while it was activated. This resulted in an average liquid retention of 0.179 μL, with a coefficient of variation of 3%. In contrast, in the conventional pelleting experiments (red bars), a droplet containing 1×10^6 beads was moved across the magnet while the densifying electrode was not activated, resulting in a total retention of ~ 0.02 μL, with a coefficient of variation of 30%, comprising both beads and liquid. In these experiments, 0.01% Tetronic 90R4 in PBS and 130 V_{RMS} were used.

Table S1. On-chip pelleting on DMF devices from 4 μL droplets without actuating the densifying electrode. For each condition, at least 6 trials were run. An unsuccessful pelleting means that the beads were displaced from the magnetic lens location by the droplet.

Number of beads	Water + 0.5% (w/v) Tetronic 90R4		PBS + 0.01% (w/v) Tetronic 90R4	
	Successful	Unsuccessful	Successful	Unsuccessful
1×10^6	✓		✓	
500×10^3	✓			✓
250×10^3		✓		✓

Table S2. Values used in the mathematical model described in Note S1.

Variable/Parameter	$\sigma\ (mN/m)$	$w\ (mm)$	$d\ (mm)$	$h\ (mm)$	k
Value	30 – 70	1.5 – 3.5	0.02 – 1.4	0.18	0.5 – 0.9

Video S1: This video demonstrates the concept of parallel loading of sub-volumes of 100 µL liquid into DMF and the subsequent concentration of Simoa beads, illustrating steps 1-13 in Figure 2a in the main text. In some (but not all) of the steps, white overlays are shown (generated automatically in the MicroDrop software) to indicate which electrodes are being engaged.

Video S2: This video shows the concept of stepwise loading of 100 µL liquid into DMF and the subsequent concentration Simoa beads, illustrating the steps in Figure 3a of the main text.

Note S1: Mathematical model for liquid retention on the bead densifying electrode

A mathematical model was developed to predict the effects of variations in experimental conditions on the pressure difference Δp experienced by a droplet as it is dispensed onto a densifying electrode. See main text for details of the system. An inequality condition can be expressed as follows:

$$(p_2 - p_1) > 0 \quad \text{[S1]}$$

where p_2 and $p_1$represent the inner pressures in the necking region and daughter droplet, respectively (see Figure 4a in the main text). The inequality condition can be further derived using the Laplace equation and the principal radii of an elongated droplet:

$$\sigma\left[\left(\frac{1}{r_2^{'}} + \frac{1}{r_2^{''}}\right) - \left(\frac{1}{r_1^{'}} + \frac{1}{r_1^{''}}\right)\right] > 0 \quad \text{[S2]}$$

Where σ represents the interfacial tension, and $r^{'}$ and $r^{''}$ are the principal radii calculated for each region of the elongated droplet. The expressions for the principal radii are given by the following equations:[1]

$$r_2^{'} = \frac{h}{-2\cos\theta_0} \quad ; \quad r_2^{''} = \frac{-L_1}{Z^2} \quad \text{[S3]}$$

$$r_1^{'} = \frac{h}{-\cos\theta_0 - \cos\theta_V} \quad ; \quad r_1^{''} = \frac{d}{2} \qquad [S4]$$

where h and d are the inter-plate gap height and the diameter of the densifying electrode, respectively, and θ_0 and θ_V are the liquid-solid contact angles when the actuation voltage is 0 and V, respectively. The assumption is that the actuation voltages are high enough for the liquid/gas interface to follow the circular shape of the electrode. To find Z, the following equation is solved:[1]

$$aZ^4 + bZ^2 + c = 0 \qquad [S5]$$

where, a, b, and c are:

$$a = L^4 + 2L^2L_1^2 + 2L^2L_2^2 + L_1^4 - 2L_1^2L_2^2 + L_2^4 \qquad [S6]$$

$$b = -4L^2L_2^2 - 4L^2L_1L_2 - 4L_2^4 + 4L_1L_2^3 + 4L_1^2L_2^2 - 4L_1^3L_2 \qquad [S7]$$

$$c = 4L_1^2L_2^2 - 8L_1L_2^3 + 4L_2^4 \qquad [S8]$$

L_1, L_2, and L specify the shape of the droplet, which, for our electrode design, are:

$$L_1 = \frac{w}{2} \quad ; \quad L_2 = \frac{d}{2} \quad ; \quad L = w \qquad [S9]$$

where w is the width of the driving electrodes.

Eq. [S2] can be further rewritten as:

$$\sigma\left[-\frac{2Z^2}{w} - \frac{2}{d} + \frac{k}{h}\right] > 0 \qquad [S10]$$

where k is the contact angle parameter:

$$k = \cos\theta_V - \cos\theta_0 \qquad [S11]$$

A higher value of the left-hand side of Eq. [S10] indicates a higher probability of breakup for a given electrode diameter.

Since Eq. [S5] results in four solutions for Z, Eq. [S10] gives four values for Δp. Since higher Δp values indicate a higher chance of droplet breakup, we focus on the solutions that yield the lowest Δp to identify the most interesting values and to plot them on Fig. 4e-g in the main text. Table S2 lists the values used for these plots.

Supplementary References

1 E. Samiei and M. Hoorfar, *Journal of Micromechanics and Microengineering*, 2015, **25**, 55008.
